# The Role of Nanobubbles in Protein Unfolding during Electrothermal Supercharging

**DOI:** 10.1021/jasms.4c00472

**Published:** 2025-03-11

**Authors:** George Joseph, Bincy Binny, Andre R Venter

**Affiliations:** Department of Chemistry, Western Michigan University, Kalamazoo, Michigan, 49008-5413, United States

**Keywords:** nanobubbles, electrothermal supercharging, protein unfolding, Tesla valve, flow regime switching

## Abstract

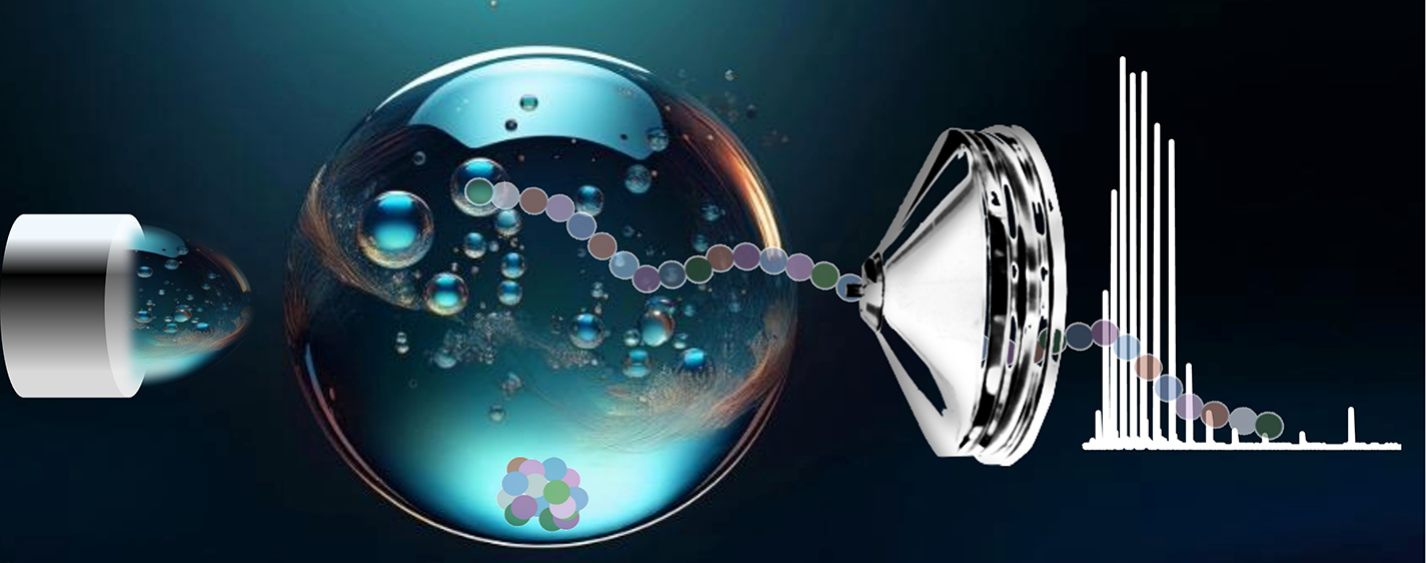

Nanobubbles (NBs) are tiny gas cavities with diameters around 200 nm that remain stable in solution due to their unique properties, including low buoyancy and negative surface charges. Ammonium bicarbonate (ABC) is an alternative buffer to commonly used ammonium acetate during protein analysis by electrospray ionization (ESI) mass spectrometry. The addition of ABC under high voltage and temperature conditions can lead to protein unfolding, a phenomenon termed electrothermal supercharging (ETS). The role of CO_2_ bubbles in ETS has been hypothesized and disputed. The solution stability of NBs allows for the direct observation of their effects on protein charge states and unfolding, providing insights into the potential role of CO_2_ bubbles during ETS. A novel method based on flow regime switching using a Tesla valve is employed to generate stable nanobubbles in solution. NBs were also created by sonication and pressure cycling. Nitrogen and carbon dioxide nanobubbles, when produced by flow regime switching and by pressure cycling, unfold proteins such as cytochrome c and ubiquitin but not to the same extent as with ABC addition to the ESI working solution. Complete unfolding of these proteins by NBs only occurs when the ammonium ion is also present in solution. Myoglobin, known to be less structurally stable, does unfold completely under NB influence. Further, amino acids, previously shown to provide stability to proteins under ETS conditions, also prevent unfolding when NBs are present, providing additional support for the role of gas bubbles during ETS.

## Introduction

Ultrafine bubbles, also known as nanobubbles (NBs), are tiny gas cavities with diameters around 200 nanometers. Unlike microbubbles, NBs do not merge and burst at the liquid surface. Instead, they remain stable in solution for long periods.^1^ This stability is due to features such as increased Brownian motion, low buoyancy, and negative surface charges.^2^

NBs exhibit a strong affinity for hydrophobic surfaces,^3^ have exceptionally large wetting angles,^4^ generate free radicals,^5^ and maintain high internal pressure.^6^ An essential characteristic of NBs is their surface electrical charge, which is represented by the zeta potential. The zeta potential varies depending on the type of gas and solvent and is typically negative. In water, O_2_, and N_2_ NBs have zeta-potentials of −34 to −45 mV and −29 to −35, respectively, while CO_2_ has zeta potential of −33 mV.^7^ Bulk nanobubble in water is stabilized by balancing the surface tension of a shrinking microbubble with the electrostatic repulsion of hydroxyl ions, which initially adsorb onto the precursor microbubble surface before shrinking.^8^ The surface hydroxyls lead to the formation of an electrical double layer contributing to the long-term lifetime of the NBs by preventing coalescence.^9^ These characteristics make NBs very promising for use in a wide range of cutting-edge scientific domains,^2,10,11^ and they find widespread application such as wastewater treatment,^12^ surface cleaning,^13^ drug delivery,^14^ and tumor destruction.^15^

Electrospray ionization mass spectrometry (ESI-MS) is a useful tool for the analysis of small to very large polar molecules and is essential in proteomics,^16^ metabolomics,^17^ lipidomics,^18^ and numerous other fields.^19^ Additives are frequently used as part of the sample working solution, especially when proteins are analyzed in their native state. The most common additive used during native mass spectrometry is ammonium acetate.^20^ Ammonium acetate is, however, not a buffer at neutral or physiological pH,^21,22^ and ammonium bicarbonate (ABC) has been suggested as an alternative. It was discovered that when ABC is used as buffer with ESI-MS under high voltage and temperature conditions, significant unfolding of protein molecules occurs. The formation of high-charge-state protein ions with electrospray from purely aqueous ammonium bicarbonate solutions at neutral pH, where the proteins have native or native-like conformations prior to ESI droplet formation, was first demonstrated in 2012.^23^ The term electrothermal supercharging (ETS) was coined to refer to the unfolding of native protein from ammonium bicarbonate solutions under conditions of high spray potential and high temperature. Konermann et al. hypothesized that CO_2_ bubble formation from the protonated bicarbonate anion could be responsible for the unfolding effect by increasing the hydrophobic surface area of protein containing droplets.^24^ This hypothesis was challenged by the observation that the effectiveness of the ETS depends on the position of the anion in the reverse Hofmeister series. The Hofmeister series is an ordering of anions and cations based on the tendency for denaturation or aggregation of proteins.^25^ A reverse Hofmeister series dependence on unfolding or stabilization is observed when proteins are positively charged.^26^

Moreover, other solutions bubbled with various gases did not cause unfolding.^27^ While this destabilization of the protein by bicarbonate is the accepted explanation currently, we showed recently that the simple addition of stabilizing reagents such as proline and imidazole can reduce the extent of apparent protein unfolding and supercharging in ammonium bicarbonate solution during ESI-MS analyses.^28^ Importantly our results provide evidence against thermal unfolding during electrothermal supercharging, a tenet of the unfolding-according-to-the-reverse-Hofmeister-series hypothesis. Recently it was again suggested that NH_4_HCO_3_ decomposed into CO_2_ and formed “microbubbles” within the microdroplets of ESI.^29^ It was suggested that these microbubbles could act as a direct internal CO_2_ source, speeding up the reaction between amines and carbon dioxide. In our work on improving protein analysis by desorption electrospray ionization (DESI-MS) we also considered CO_2_ bubbles produced by cavitation during droplet-surface collisions as a possible explanation for how ABC increases protein signal intensities.^30,31^ These points motivate us to reopen the possibility of the formation of CO_2_ bubbles during ETS as a driver for the observed protein charge state increases.

**Figure 1:**
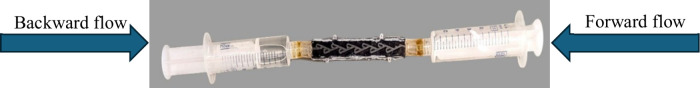
Nanobubble generation by flow regime switching using a Tesla valve.

We recently showed that nanobubbles can be deliberately added to ESI working solutions to great beneficial effect, increasing the signal intensities for all compound classes studied including protein.^32^ In this work we generate CO_2_ and N_2_ NBs using a novel method based on flow regime switching by the Tesla valve.^33,34^ The forward flow of the sample through the Tesla valve creates turbulent flow inside the valve, leading to the evolution of microbubbles from the gas-saturated solution, which are then further broken down into nanobubbles. In the Tesla valve, turbulence is achieved at low Reynolds Numbers with lower flow resistance and low energy consumption. Flow in the opposite direction is laminar due to the unimpeded flow pattern in this direction (Figure 1). Two other batch preparation methods for lab-scale nanobubble production were also used for comparison, namely, sonication^35^ and pressure cycling.^36^

With the addition of solution-stable NBs to working solutions in ESI we can directly study the potential role of CO_2_ bubbles on protein unfolding in ETS without needing to infer their existence.

## Experimental Section

### Materials

A 99.5% pure ethyl alcohol, bovine heart cytochrome C (cyt c, 95% purity), ubiquitin (Ub, 98% purity), myoglobin (Mb, ≥90% purity), BioUltra grade ammonium bicarbonate (ABC), ammonium acetate (NH_4_OAc), ammonium hydroxide (NH_4_OH), sodium acetate (NaOAc) proline, hydroxyproline, imidazole (98% purity), and formic acid (FA, ≥98%) were purchased from Sigma-Aldrich (St. Louis, MO). The protein stock solution was prepared at 160 μM concentration. Solvent system stock solutions at 1.0 M each of NH_4_HCO_3_ (ABC), NH_4_OAc, NH_4_OH, and NaOAc were prepared in Milli-Q water obtained from a Thermo-Barnstead Water Polisher (Thermo Scientific, Waltham, MA, USA). Protein standards were diluted to 10 μM in various solvent systems and are referred to as controls. Similar protein solutions were prepared where the water fraction was enriched in NBs.

### Nanobubble Generation

A 3D-printed Tesla valve with a length-to-depth ratio of 21, created with a Phrozen Sonic Mini 8K LCD Resin 3D printer using Siraya Tech Blu 3D printer resin (San Gabriel, California), was employed to generate NBs. The print file was based on a plan published by Yunhao Bao et al.^37^ Carbonation of a 3% ethanol solution was obtained by using a SodaStream (Mount Llaurel, New Jersey) soda water maker. Carbonated 3% ethanol solution was used for the generation of CO_2_ NBs. Similarly, a 3% ethanol solution was sparged with 99.999% N_2_ (Airgas, Gwinnett, Georgia) for 5 min at a tank pressure of 50 psi and volumetric flow rate of 5.8 L/min, which is then used for the generation of N_2_ NBs. NB generation was optimized to 12 cycles. One cycle comprises a forward and backward directional flow, as shown in Figure 1. CO_2_ NBs created by this method were used throughout the manuscript, except in Figure 5 where N_2_ NBs were used and in Figure 4 where CO_2_ NBs were also created by sonication and pressure cycling for comparison, under conditions previously optimized.^34^

NBs generated by pressure cycling^36^ required a 5 mL carbonated sample to undergo 60 cycles in a 10 mL polypropylene syringe.

Nanobubbles produced by the sonication method^38^ were irradiated for 5 min at a frequency of 10 kHz using a Misonix Ultrasonic Liquid Processor XL-2020 probe (Misonix Farmingdale, NY).

### Nanobubble Characterization

The size distributions and bubble concentrations formed after the treatment with each previously optimized NB generation method were determined by PMX-230-Z-TWIN-488/640 Laser Zeta View Nanoparticle Tracking Analysis system (NTA) (Particle Metrix, Ammersee, Germany).^34^ Measurement settings were: Camera sensitivity (82), Shutter (110), Cell temperature (24.09 °C). The video analysis parameters were Maximum area (1000), Minimum area (10), and Minimum brightness (18). Characterization data is presented in Table 1.

**Table 1:** Physical characterization of CO_2_ nanobubbles under optimized conditions for each of the generation methods using the Nanoparticle Tracking Analysis system (NTA)

	Weighted Average Size		Concentration		
Method	Diameter (nm)	RSD%	(NBs/ml)	RSD%	Zeta Potential (mV)
Pressure Cycling	143	3.58	2.61E9	19.19	–21.27
Sonication	99	11.00	2.99E8	14.04	–36.17
Tesla Valve	110	8.27	3.79E8	7.43	–33.30

### Mass Spectrometry

Experiments were performed on an LTQ linear ion trap mass spectrometer (Thermo Scientific, Waltham, MA). Electrospray ionization was produced using either a Thermo Ion Max source or a home-built microelectrospray emitter made from a Swagelok T-piece and two pieces of coaxial fused silica capillary tubing. The outer capillary (for sheath gas) was approximately 20 mm in length with an outer diameter of 360 μm and an inner diameter of 250 μm. The internal capillary (for solvent) had an outer diameter of 150 μm and an inner diameter of 50 μm. The solvent capillary extended through the T-piece and was connected to a syringe pump, which delivered the working solutions for direct infusion. Spray potential was applied to the liquid junction of a stainless-steel syringe needle which delivered solvent at a flow rate of 5 μL/min, with N_2_ as nebulizing gas at 100 psi. LTQ capillary and tube lens voltages were set at 30 and 135 V for the analysis of the protein.

### Data Analysis

Mass spectra were collected and viewed in the Xcalibur Qual Browser (2.0.7). Thirty scans of direct infusion of three independent samples were collected for each solvent system of protein.

## Results and Discussion

The electrothermal supercharging (ETS) phenomenon was investigated by evaluating the charge states obtained for a 10 μM cyt c solution in 100% water or water containing additives including 100 mM ABC, 100 mM NH_4_OAc, or 100 mM NH_4_OH at different source temperatures and spray voltages. For analyzing the NB effect, the water in every solvent system was substituted with CO_2_ NB-enriched solutions.

**Figure 2:**
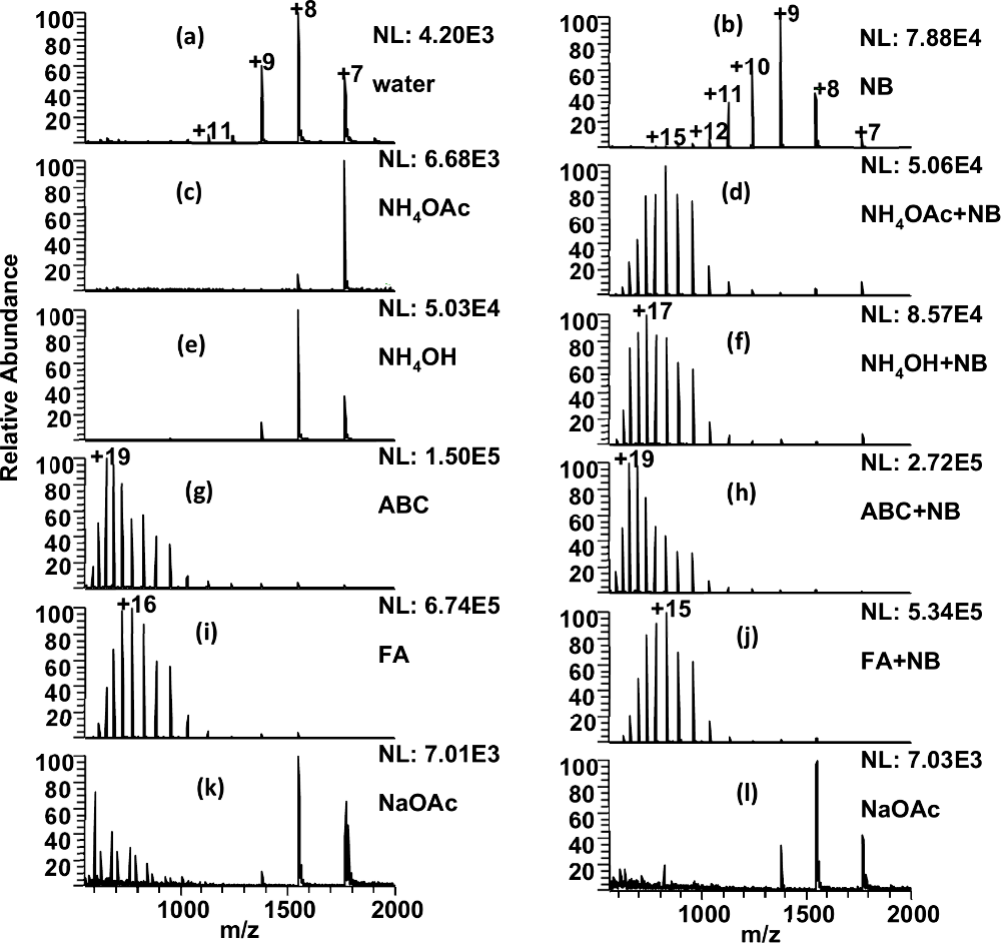
10 μM Cytochrome c in (a) 100% water, (b) 100% water + NB, (c) 100 mM NH_4_OAc, (d) 100 mM NH_4_OAc + NB, (e) 100 mM NH_4_OH, (f) 100 mM NH_4_OH + NB, (g) 100 mM ABC, (h) 100 mM ABC + NB, (i) 0.2% FA, (j) 0.2% FA + NB, (k) 1 mM NaOAc, (l) 1 mM NaOAc + NB. All spectra on the right are with CO_2_ NBs. The normalization level (NL) indicates the absolute signal intensity.

The representative spectra of cyt c in 100% water, aqueous 100 mM ABC solution, and 100% CO_2_ NB solution at an ion transfer tube temperature set to 250 °C and a spray potential of 4 kV applied to the emitter are shown in Figure 2.

The mass spectrum for cyt c in its native state from pure H_2_O with no additive is shown in Figure 2a. The mass spectra are described by using the metrics of highest intensity charge state (HICS) for the most abundant protein ion in the envelope, the highest observed charge state (HOCS) with a S/N > 3, and the weighted average charge state. In Figure 2a, cyt c has an HICS at z = +8, an HOCS at z = +11, and an average charge state of z = 8.2. Adding ammonium acetate (Figure 2c) and ammonium hydroxide (Figure 2e) to the working solution narrows the charge state distribution, with an HICS at z = +7 and an HOCS at z = +8, indicating folded conformations for cyt c. When analyzed from a solution containing 100 mM ammonium bicarbonate solution, the HICS increased to +19 and the HOCS increased to +22, with an average charge state of z = 16.8 (Figure 2g). This shift in charge state to higher values with ABC, at high temperature and spray voltage, is what is commonly referred to as ETS.^24^ Formic acid also readily unfolds the protein to a slightly lesser extent. Here the HICS for the control experiment was z = +16 and a HOCS of z = +21. The addition of CO_2_ NBs does not cause further increases to charge states to take place for either ABC or formic acid-containing solvent systems.

When the cyt c is sprayed from pure water enriched with CO_2_ NBs the protein envelope shifts to higher charge states with an average charge state of z = 9.6. The HICS and HOCS also increase to z = +9 and z = +15, respectively, as shown in Figure 2b. While the protein unfolds under the presence of CO_2_ NBs in pure water, it does so less extensively than observed for ETS using ABC. Surprisingly, in the ammonium acetate (Figure 2d) and ammonium hydroxide (Figure 2f) solution, when CO_2_ NBs are also present, complete unfolding is observed, and the mass spectra resemble that of the ABC-induced ETS result. This holds true for solvent systems containing any of the ammonium salts together with NBs. When NBs are added to the ammonium acetate solution, the HICS increases from z = +7 to z = +15, while the HICS of the protein analyzed with ammonium hydroxide (Figure 2f) increases from z = +8 to z = +17 when NBs are added.

The importance of the ammonium ion was further demonstrated in Figure 2(k,l) where the sodium acetate salt was used instead of the ammonium. Only a small increase in average charge state occurs when NBs are added, while the HICS remains at z = +8 with and without NBs (Figure 2(i,j)). These results indicate that in addition to CO_2_ NBs, ammonium ions are also necessary for extensive cyt c supercharging.

Besides contributing to protein unfolding, nanobubbles also positively influence protein signal intensities. The magnitude of signal enhancement is solvent system-dependent. Signal increases upon NB addition ranged from an improvement to HICS intensity of nearly 2X with ABC, where the same charge state envelope resulted from both solutions, to more than 18X when NBs are added to pure water sample solutions.

We recently reported on the effects of amino acids on increasing or mitigating ETS.^28^ Among the natural amino acids, protein supercharging was significantly reduced by proline and glycine; however, imidazole provided the highest degree of noncovalent complex stabilization against ETS. Our study showed that the simple addition of stabilizing reagents such as proline and imidazole can reduce the extent of apparent protein unfolding and supercharging in ammonium bicarbonate solution. The effects were generally in good agreement with the extensive literature available on the stabilization or destabilization of proteins by these additives in bulk solution. Results for arginine addition provide evidence against the roles of charge depletion,^39^ while the negative results for hydroxyproline,^40^ a known thermal stabilizer, cast some doubt on the role of thermal unfolding during ETS.

To further establish the link between CO_2_ NBs and ETS we also investigated the ability of a similar set of additives to modulate the extent of supercharging observed when NBs are present in solution.

**Figure 3:**
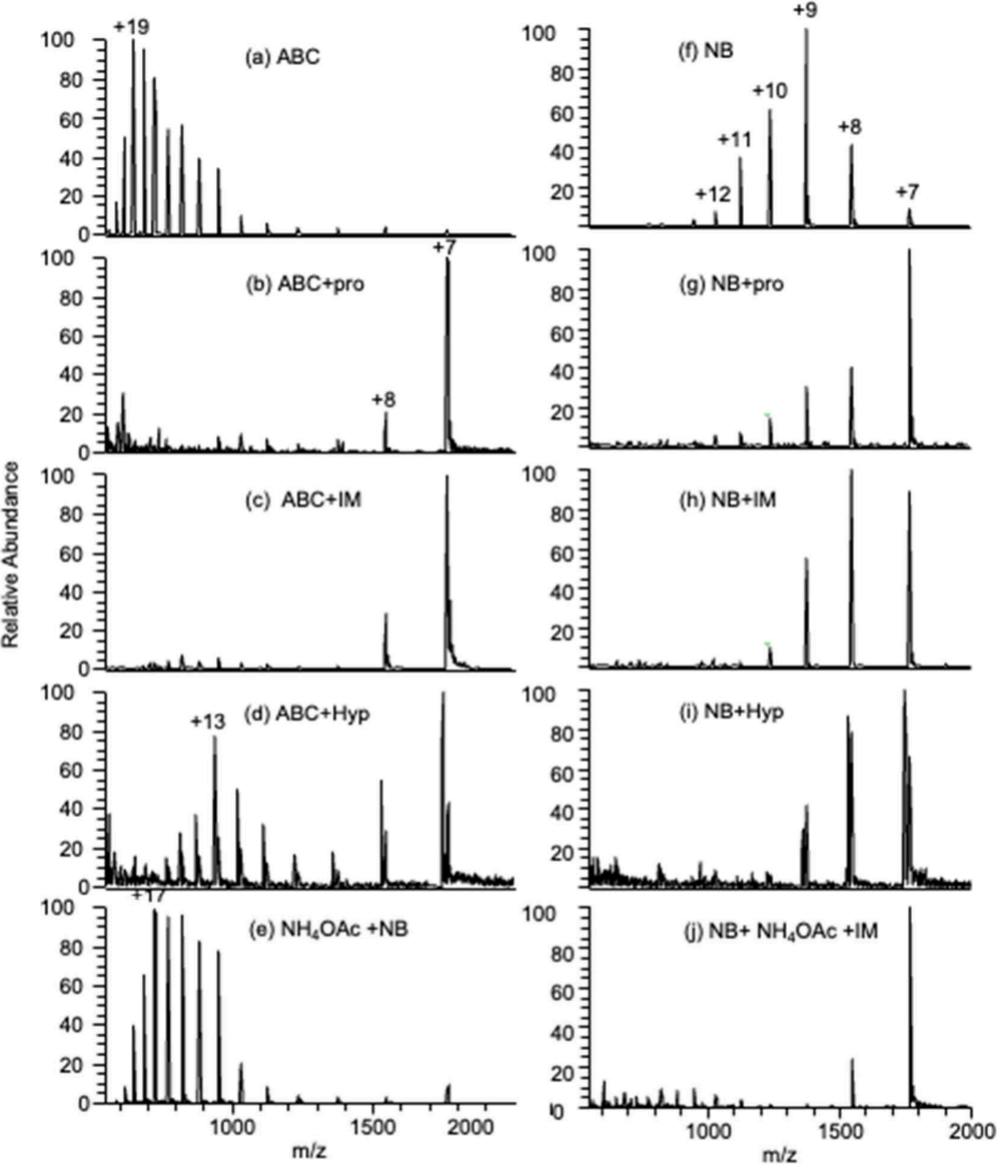
10 μM cytochrome c in (a) 10 mM ABC, (b) 10 mM ABC + 1 mM proline (Pro), (c) 10 mM ABC + 1 mM imidazole (IM), (d) 10 mM ABC + 1 mM hydroxy proline (Hyp), (e) 100 mM NH_4_OAc + NB, (f) 100% NB, (g) 1 mM proline + nanobubble solution, (h) 1 mM imidazole + NB, (i) 1 mM hydroxyproline + NB, and (j) 100 mM NH_4_Ac + 1 mM imidazole + NB solution. All spectra on the right are with CO_2_ NBs. The normalization level (NL) indicates the absolute signal intensity.

Similar to their effect with ABC addition and ETS, the stabilizing additives proline and imidazole were effective in preventing unfolding when CO_2_ NBs were present in solution. Figure 3g,h shows a shift in the HICS from z = +9 to z = +7 and z = +8, respectively, while the average charge state reduces from z = 9.6 with NBs only to z = 8.6 when proline is present and z = 8.2 with imidazole. Interestingly, just as the presence of the ammonium ion increases unfolding with NB solutions, the stabilizing effect of imidazole was also more pronounced when ammonium acetate was included in the working solution. The thermally stabilizing amino acid hydroxyproline (Hyp), which did not prevent unfolding during ETS, also did not provide any protection against unfolding in the presence of NBs.

**Figure 4:**
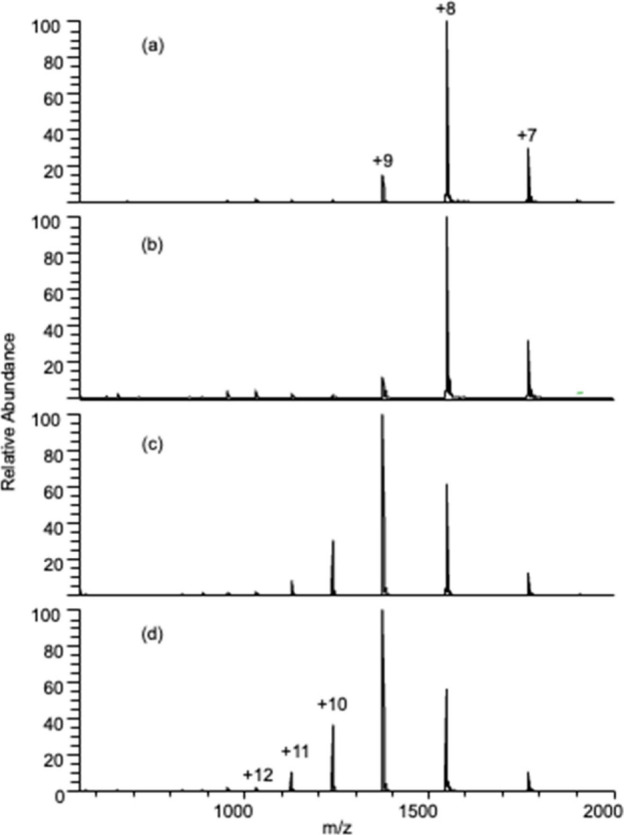
Cytochrome c analyzed by CO_2_ nanobubbles produced by three different methods. Cyt c was analyzed from (a) 100% water, (b) 100% water + NB from ultra sonication method, (c) 100% water + NB from pressure cycling method, and (d) 100% water + NB from Tesla valve.

Several methods have been reported for the laboratory-scale generation of NBs. While the work in this manuscript was performed using flow regime switching by Tesla valve we attempted to replicate the observed protein unfolding using NBs produced by other methods such as pressure cycling^36^ and sonication,^41^ as shown in Figure 4. Interestingly, while CO_2_ NBs produced by pressure cycling or flow switching with the Tesla valve create NBs that equally unfold cytochrome c, no such unfolding is observed when NBs are produced by sonication. This difference in unfolding potential cannot be described by physical measurements typically used to characterize NBs. As shown in Table 1, there are minor differences in the bubble diameters. The Tesla valve produces CO_2_ bubbles with a zeta potential nearly as high as sonication, and bubble numbers near pressure cycling, and with similar zeta potentials. This intriguing observation will require further future investigation.

**Figure 5:**
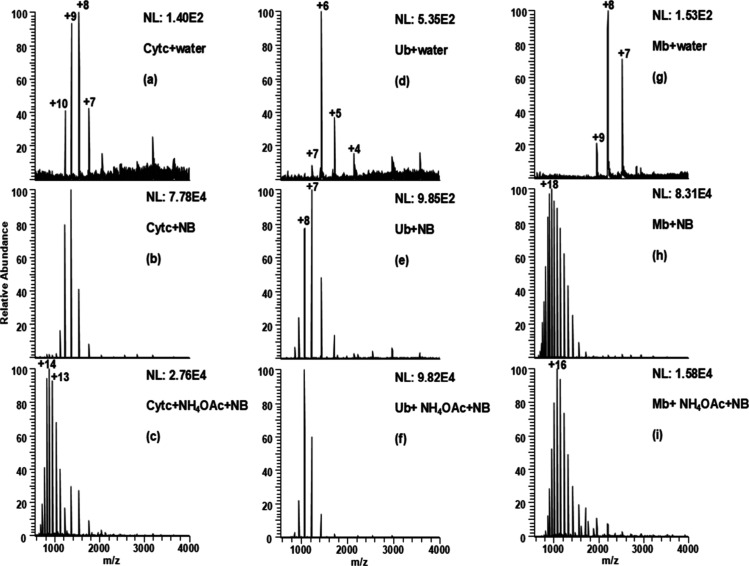
Unfolding by N_2_ nanobubbles of cytochrome c, ubiquitin, and myoglobin. Cyt c in (a) Pure H_2_O, (b) Pure water with NBs, (c) 100 mM NH_4_OAc with NBs, Ub in (d) Pure H_2_O, (e) Pure water with NBs, (f) 100 mM NH_4_OAc with NBs, Mb in (g) Pure H_2_O, (h) Pure water with NBs, and (i) 100 mM NH_4_OAc with NBs.

A high concentration of dissolved carbon dioxide, present as carbonic acid, may produce sufficient bicarbonate anion to affect the unfolding, according to the position of bicarbonate in the reverse Hoffmeister series.^27^Figure 5 shows that NBs produced from N_2_ also unfold proteins to the same extent as when CO_2_ NBs are used. The mass spectrum of cytochrome c under the effect of N_2_ NBs closely resembles that of the CO_2_ NB (Figure 5a–c). This confirms the role of NB-mediated unfolding rather than unfolding induced by the presence of the bicarbonate anion. Similar to the unfolding observed with CO_2_ NBs, while the protein unfolds with N_2_ NB addition alone, ammonium ions are also required for complete unfolding. For the results in Figure 5, those are provided by the usually stabilizing ammonium acetate additive.

The covalently bound heme group in cytochrome c plays a crucial role in enhancing structural stability.^45,46^ Similar observations were made for ubiquitin, another protein known for its compact structure and tight hydrogen bonding.^43^ This provides high structural resilience, high resistance to digestion,^42^ and stability over a wide range of pH and temperature values. For Ub, NB-induced unfolding is also not complete, and HICS increases from z = +6 to z = +7 upon the addition of N_2_ nanobubbles (Figure 5(e)). Further unfolding is observed when ammonium ions are added, as shown in Figure 5(f), where the HICS increases to z = +8.

Myoglobin, on the other hand, is a small, globular protein with a single polypeptide chain and, while it has a compact tertiary structure, lacks complex multidomain interactions, making it more susceptible to unfolding.^44^ Complete unfolding of Mb is observed when an N_2_ nanobubble-enriched solution is introduced, both with, and without, NH_4_OAc, as shown in Figure 5(h,i).

## Conclusion

We provide convincing evidence that CO_2_ NBs, potentially produced during electrothermal supercharging, can contribute significantly to the observed increases in the charge states. The evidence is based on the deliberate introduction of solution-stable CO_2_ and N_2_ NBs, leading to partial unfolding of the model proteins, cytochrome c, and ubiquitin. Complete unfolding to the same extent as with ETS requires the presence of ammonium ions, even when those ammonium ions are delivered by the native state preserving ammonium acetate additive. Proteins with weaker three-dimensional structures, such as myoglobin, unfold completely without the additional need for ammonium ions. Since unfolding also occurs when N_2_ NBs are used, the role of the internal gas–liquid interface of the NBs is confirmed over the potential effect of the bicarbonate anion. Further, unfolding can be mitigated in the presence of known stabilizing additives such as proline and imidazole, maintaining a native conformation in ammonium bicarbonate buffer as well as NB solutions during ESI analysis under ETS conditions.
